# Study of Different Chitosan/Sodium Carboxymethyl Cellulose Proportions in the Development of Polyelectrolyte Complexes for the Sustained Release of Clarithromycin from Matrix Tablets

**DOI:** 10.3390/polym13162813

**Published:** 2021-08-21

**Authors:** Víctor Guarnizo-Herrero, Carlos Torrado-Salmerón, Norma Sofía Torres Pabón, Guillermo Torrado Durán, Javier Morales, Santiago Torrado-Santiago

**Affiliations:** 1 Department of Pharmaceutics and Food Technology, Faculty of Pharmacy, Complutense University, Plaza Ramón y Cajal s/n, 28040 Madrid, Spain; victor08@ucm.es (V.G.-H.); ctorrado@ucm.es (C.T.-S.); 2 Department of Biomedical Science, Faculty of Pharmacy, University of Alcalá de Henares, Ctra Madrid-Barcelona Km 33,600, 28805 Madrid, Spain; sofia.torres@edu.uah.es (N.S.T.P.); guillermo.torrado@uah.es (G.T.D.); 3 Department of Science and Pharmaceutical Technology, Faculty of Chemical and Pharmaceutical Sciences, University of Chile, Santiago 8380494, Chile; javiermv@ciq.uchile.cl; 4 Instituto Universitario de Farmacia Industrial, Complutense University, Plaza Ramón y Cajal s/n, 28040 Madrid, Spain

**Keywords:** chitosan, carboxymethyl cellulose, clarithromycin, interpolymer complexes, hydrogel, polyelectrolyte complexes, drug delivery

## Abstract

This study investigated the combination of different proportions of cationic chitosan and anionic carboxymethyl cellulose (CMC) for the development of polyelectrolyte complexes to be used as a carrier in a sustained-release system. Analysis via scanning electron microscopy (SEM) Fourier transform infrared spectroscopy (FTIR), differential scanning calorimetry (DSC), and powder X-ray diffraction (PXRD) confirmed ionic interactions occur between the chitosan and carboxymethyl cellulose chains, which increases drug entrapment. The results of the dissolution study in acetate buffer (pH 4.2) showed significant increases in the kinetic profiles of clarithromycin for low proportions of chitosan/carboxymethyl cellulose tablets, while the tablets containing only chitosan had high relaxation of chitosan chains and disintegrated rapidly. The Korsmeyer–Peppas kinetic model for the different interpolymer complexes demonstrated that the clarithromycin transport mechanism was controlled by Fickian diffusion. These results suggest that the matrix tablets with different proportions of chitosan/carboxymethyl cellulose enhanced the ionic interaction and enabled the prolonged release of clarithromycin.

## 1. Introduction

Sustained-release systems prolong the time taken for a drug to reach maximum plasma concentrations, allowing for a reduced frequency of dosage, and they are associated with decreased adverse side effects and good safety profiles. Matrix tablets have been widely used by the pharmaceutical industry for drug delivery mainly due to their simple preparation process and low production costs.

Since the development of polyelectrolyte complexes (PECs), there has been growing interest in their use in the control and modulation of drug release from hydrogel matrices. Electrostatic attractions between the ionized amino (NH_3_^+^) group of chitosan and the carboxylic (COO^−^) groups of the anionic polymer are the main interactions in the formation of polyelectrolyte hydrogels with a high swelling ratio in the dissolution medium [1,2].

Chitosan (CS) is the most widely used cationic polymer; it has good biocompatibility and is nontoxic and biodegradable [3]. Its molecular weight control and degree of deacetylation also allow its use in PECs for the sustained release of drugs [4,5].

Natural anionic polymers have been extensively used for the development of PECs [5,6,7,8]. However, anionic cellulose derivatives such as hypromellose phthalate [4] and carboxymethyl cellulose sodium [9] show greater control of the molecular weight and the number of carboxylic groups (COO^−^). Carboxymethyl cellulose sodium (CMC) is a cellulose derivative with good solubility in water. CMC has a linear polymer chain and displays polyelectrolyte behavior due to the weak acidic groups in its molecular backbone [9]. The degree of substitution, which is determined by the number of carboxymethyl groups per unit of CMC, has been widely studied and is one of the main factors affecting its physicochemical behavior [9,10]. Recent studies have shown that the development of matrix tablets containing mixtures of CS and anionic polymers self-assemble during the compression process [6,7].

The administration of PECs with proton pump inhibitors increases gastric pH values to over pH 4.0 [11,12], indicating that in combination treatments with proton pump inhibitors, the use of matrix tablets with interpolymer complexes allows for the sustained release of different model drugs. The pKa of CS is about 6.5, and thus, in a medium with a pH of 4.2, the CS is protonated and produces positively charged NH_3_^+^ [1], whereas the pKa of CMC is about 3.5 [13], meaning it is insoluble in acid and forms an insoluble network structure [6]. Although it has a similar pH to that obtained with proton pump inhibitors (acetate buffer pH4.2), the CMC presented anionic charges (COO^−^) that interact with the ionized amino group of chitosan (NH_3_^+^) and form CS/CMC complexes. 

Clarithromycin (CL) is an oral broad-spectrum antibiotic that is effective in the treatment of respiratory tract infections. It is also the first line of treatment for *Helicobacter pylori* [11]. CL is poorly soluble (Class II), and its solubility is dependent on the pH of the medium due to its weak base character with a pKa of 8.9 [14]. The solubility of CL increases as the pH of the dissolution medium decreases. CL is also unstable in an acidic medium (pH 1–2) but stable at a pH above 4 [15]. The use of omeprazole with CL PECs allows for sustained release systems that maintain high levels of CL in the gastric environment [12,15].

The presence of (COO^−^) groups, such as in CL, and their pKa favor their inclusion in CS/CMC interpolymeric complexes at a pH close to 4.0 [15]. The use of different kinetic models may be important for understanding drug release from PECs [16,17].

The aim of the present work was to study the different electrostatic interactions between the cationic chains of CS and the carboxylic groups of CMC at pH 4.2. FTIR, DSC and X-ray diffraction techniques were employed to study the polymer/polymer interactions between CS/CMC and the drug/polymer interactions for the development of different PECs. The studies of CL sustained release at pH 4.2 and its fit to the Korsmeyer–Peppas kinetic model were used to select the different dissolution profiles of CL in this dissolution medium (acetate buffer pH 4.2).

## 2. Materials and Methods

### 2.1. Materials

High-molecular-weight chitosan (CS) (MW 310–375 kDa and deacetylation degree > 75%) and high-viscosity carboxymethyl cellulose sodium (CMC) (MW 1500–2500 Da and substitution degree of 70%, equivalent to 0.7 carboxymethyl groups per anhydroglucose unit) were purchased from Sigma-Aldrich (Saint Louis, MO, USA). Clarithromycin (CL) was provided by Normon Pharmaceutical Co., Ltd. (Madrid, Spain). All other reagents and chemicals used were of analytical grade.

### 2.2. Methods

#### 2.2.1. Preparation of Formulations

Clarithromycin, chitosan, and carboxymethyl cellulose raw materials were used as references in Fourier transform infrared (FTIR), powder X-ray diffraction (PXRD), differential scanning calorimetry (DSC), and dissolution studies. The physical mixture (PM) of clarithromycin:(chitosan/carboxymethyl cellulose *w*/*w*) PM-CL:(CS:CMC) 80:(18:2) was formulated by mixing 1000 mg of clarithromycin, 225 mg of chitosan, and 25 mg of carboxymethyl cellulose, whereby the drug and polymers had previously been sieved through a 0.45 mm mesh.

To evaluate the influence of chitosan, clarithromycin/chitosan hydrogel tablets were prepared without carboxymethyl cellulose: CL:(CS) at 80:(20), 60:(40), and 40:(60). CS/CMC interactions were studied with the following proportions of polyelectrolyte complexes (PEC) CL:(CS:CMC) at 80:(18:2), 80:(10:10), 40:(54:6), and 40:(30:30).

The hydrogel tablets were produced by sieving 1000 mg of clarithromycin and different proportions of chitosan through a 0.45 mm mesh and mixing these materials in a ceramic bowl using a polymeric spatula, which were then directly compressed on a single punch tableting machine (Bonal type A; Barcelona, Spain) using a 6 mm diameter punch. Tablet weights were adjusted to 50 mg and 40–80 N of hardness.

PEC tablets of clarithromycin were prepared by mixing 1000 mg of the active ingredient with the different proportions of chitosan and carboxymethyl cellulose in a ceramic bowl, whereby the mixed components had first been sieved through a 0.45 mm mesh. The PEC tablets were prepared using a single punch tableting machine (Bonal type A; Barcelona, Spain) with a 6 mm diameter punch. Tablet weights were adjusted to 50 mg and 40–80 N of hardness.

#### 2.2.2. Scanning Electron Microscopy (SEM) Studies

The samples were mounted on an aluminum sample mount. After coating with a thin layer of gold-palladium, the hydrogel samples were analyzed via SEM using a Jeol^®^ 6400. All micrographs were the product of secondary electron imaging used for surface morphology identification at different magnifications and an accelerating voltage of 20 kV. 

#### 2.2.3. Fourier Transform Infrared Spectroscopy (FTIR)

The different samples were prepared by pressing 2 mg of powder with 200 mg of potassium bromide at 10 T (Carver hydraulic press Model C-3912; Carver Inc., Wabash, IN, USA). Absorbance spectra were measured using a Fourier transform infrared spectroscopy (FTIR) spectrophotometer (Perkin Elmer 1600 FTIR spectrophotometer; Perkin Elmer Inc., Waltham, MA, USA). The spectra were obtained at a 2 cm^−1^ resolution with an average of 32 scans, with air as background. The infrared region was analyzed in the range of 4000–500 cm^−1^.

#### 2.2.4. Differential Scanning Calorimetry (DSC)

DSC thermograms were obtained using an automatic thermal analyzer system (Mettler Toledo TC 15, TA controller). The temperature was calibrated using an indium reference standard for calibration (transition point 156.60 XC). All dried samples were accurately weighed into aluminum pans, hermetically sealed with aluminum lids, and heated from 25 to 250 °C at a rate of 10 °C/min under constant purging with dry nitrogen at 30 mL/min. An empty pan was sealed and used as a reference with the same sample conditions.

#### 2.2.5. Powder X-ray Diffraction (PXRD)

PXRD patterns were recorded on a Philips X’Pert-MPD X-ray diffractometer (Malvern Panalytical; Almelo, Netherlands) in the CAI (Centro de Asistencia a la Investigación, Universidad Complutense, Madrid, Spain). The samples were radiated using a monochromatized CuKα radiation (λ = 1.542 Å) then analyzed in the range of 5–50°(2θ) at a step size of 0.04° and a time of 1 s per step. The voltage was 30 kV with a current of 30 mA.

#### 2.2.6. In Vitro Drug Release

These studies evaluated the hydrogel tablets of CL:(CS) 80:(20), 60:(40), and 40:(60), and the PEC tablets of CL:(CS:CMC) 80:(18:2), 80:(10:10), 40:(54:6), and 40:(30:30).

The evaluation was conducted in a dissolution bath (Vankel^®^ VK 700). A USP Apparatus 2 (paddle) was set up at 37 °C, with a rotational speed of 50 rpm and 500 mL of 0.1 N HCl or acetate buffer (pH 4.2). The pH 4.2 dissolution test method for clarithromycin is established by the United States Pharmacopeia (USP42-NF32, 2019). A sample of 5 mL was removed at time points of 15, 30, 45, 60, 90, 120, 180, 240, 300, 360, 420, and 480 min and filtered through a 0.45 μm filter (Acrodisc^®^; New York, NY, USA).

The quantity of clarithromycin was determined using the HPLC method, consisting of a UV detector (Jasco UV-1575 Intelligent UV/VIS Detector), a pump (Jasco PU-1580 Intelligent HPLC pump), a degasser (Jasco DG-2080-53), and an automatic injector (Gilson^®^ 231 XL Sampling Injector). The HPLC method was developed from previous studies with the following changes [11]: The selected wavelength was 210 nm. A C18 column (4.6 mm × 15 cm) was used with a particle size of 5 μm, and the temperature was maintained constant at approximately 60 °C (Pickering Laboratories CHX700 Column Temperature Controller). The flow rate was 1.0 mL per minute. The mobile phase consisted of a mixture of methanol and 0.079 M monobasic potassium phosphate (65:35, *v*/*v*), and the pH was adjusted to 4.2 with phosphoric acid. The cumulative amount of clarithromycin released from the system was determined from the appropriated calibration curve. The determination at each time point was performed in triplicate, and the error bars on the graphs represented the standard deviation.

To investigate the effect of polyelectrolyte complex formation on the release of clarithromycin more precisely, the results were analyzed according to the Korsmeyer-Peppas Equation for $M_{t}/M_{\infty }$ < 0.6, which can be expressed as the following Equation [17]:

(1) $$M_{t}/M_{\infty }=k_{d}\;t^{n}$$

where $M_{t}/M_{\infty }$ is the fractional drug released at time *t* (h) from the total amount released $M_{\infty }.$ *K_d_* (min^−1^) is the kinetic dissolution constant and *n* is a diffusional exponent characteristic of the release as a function of time *t*. For drug release from tablets, which contains hydrophilic polymers, the exponent *n* is the diffusional constant that characterizes the drug release transport mechanism. When *n* = 0.5, a Fickian diffusion process was observed, where drug diffusion through the polymeric matrix is the dominant release mechanism. When *n* values are between 0.5 < *n* < 1, drug diffusion occurs via anomalous transport (non-Fickian). In anomalous diffusion, it is assumed that the mechanism of CL release is a combination of swelling, erosion, and diffusion. When *n* = 1, Case II transport or zero-order release kinetics could be observed. An *n* value of about 1.0 indicates that polymer relaxation, polymer dissolution, or tablet erosion are the dominant mechanisms [16,18].

For the mathematical evaluations, we characterized drug release kinetics by fitting standard release to zero-order, Higuchi, and first-order models [19,20].

(2) $$M_{t}/M_{\infty }=K_{0}\;t\;\mathrm{zero}-\mathrm{order}\;\mathrm{model}$$

(3) $$M_{t}/M_{\infty }=K_{H}\;t^{0.5}\;\mathrm{Higuchi}\;\mathrm{model}$$

(4) $$Ln\;(M_{t}/M_{\infty })=-K_{1}\;t\;\mathrm{first}-\mathrm{order}\;\mathrm{model}$$

where $M_{t}/M_{\infty }$ is the fractional drug released at time *t* (min) from the total amount released $M_{\infty }$. *K_H_*, *K*_0_, and *K*_1_ (min^−1^) are the kinetic dissolution constants for zero-order, Higuchi, and first-order kinetic models, respectively, which characterize release as a function of time *t*.

The high R^2^ values in the zero-order model indicate that polymer relaxation or tablet erosion is the dominant mechanism and is related to Case II transport for the Korsmeyer-Peppas Equation.

The high R^2^ values in the Higuchi model indicate the better fit of release data for diffusion kinetics, which are related to a Fickian diffusion process in the Korsmeyer-Peppas equation.

The high R^2^ values in the first-order model indicated swelling and erosion phenomena and could be related to non-Fickian (anomalous transport) for the Korsmeyer-Peppas equation.

## 3. Results and Discussion

### 3.1. Scanning Electron Microscopy (SEM) Characterization

**Figure 1:**
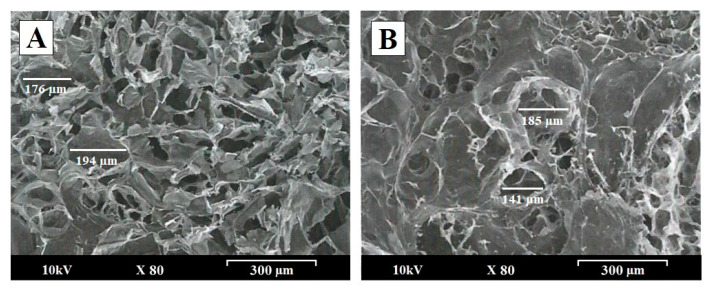
Scanning electron micrographs of CL:(CS:CMC) tablets with different (CS:CMC) proportions. PEC tablets of (**A**) CL:(CS:CMC) 80:(18:2) (*w/w*) and (**B**) CL:(CS:CMC) 40:(30:30) (*w/w*), after 1 h at pH 4.2. Original magnification is 80× and the scale bar is equal to 300 μm.

Figure 1 shows scanning electron micrographs of the surfaces of PEC tablets CL:(CS:CMC) (A) 80:(18:2) and (B) CL:(CS:CMC) 40:(30:30), after 1 h at pH 4.2. To study the influence of the proportion of CS within the interpolymer network structure, micrographs of these formulations were taken at a magnification of 80×.

After 1 h in the dissolution medium (acetate buffer, pH 4.2), the PEC tablets CL:(CS:CMC) 80:(18:2) demonstrated rapid swelling and disintegration, and these characteristics made it difficult to evaluate the changes in the surface of the tablets during dissolution studies (Figure 1A). The PEC tablets with a high proportion of carboxymethyl cellulose, CL:(CS:CMC) 40:(30:30), showed a smooth structure, with smaller pores observed on the surface (Figure 1B). The high proportion of carboxymethyl cellulose produces a dense interpolymer structure which hinders the presence of connections (channels) with the interior of the system. Highly porous structures (150–200 μm) forming connections (channels) with the interior of the system were observed in both formulations. Similar highly porous structures have been reported before for a chitosan interpolymer complex of matrix tablets, whose characteristics are related to the gel layer formed by the polymer relaxation with the absorption of the dissolution medium [21].

These differences in the surfaces can be explained by the slower repulsion between ionized chains due to a greater number of cationic moieties in formulations that have high proportions of chitosan. The presence of more anionic chains of carboxymethyl cellulose favored the formation of interpolymer complexes with fewer pores on the surface of the matrix tablets [22].

### 3.2. FTIR Spectroscopy Analysis

**Figure 2:**
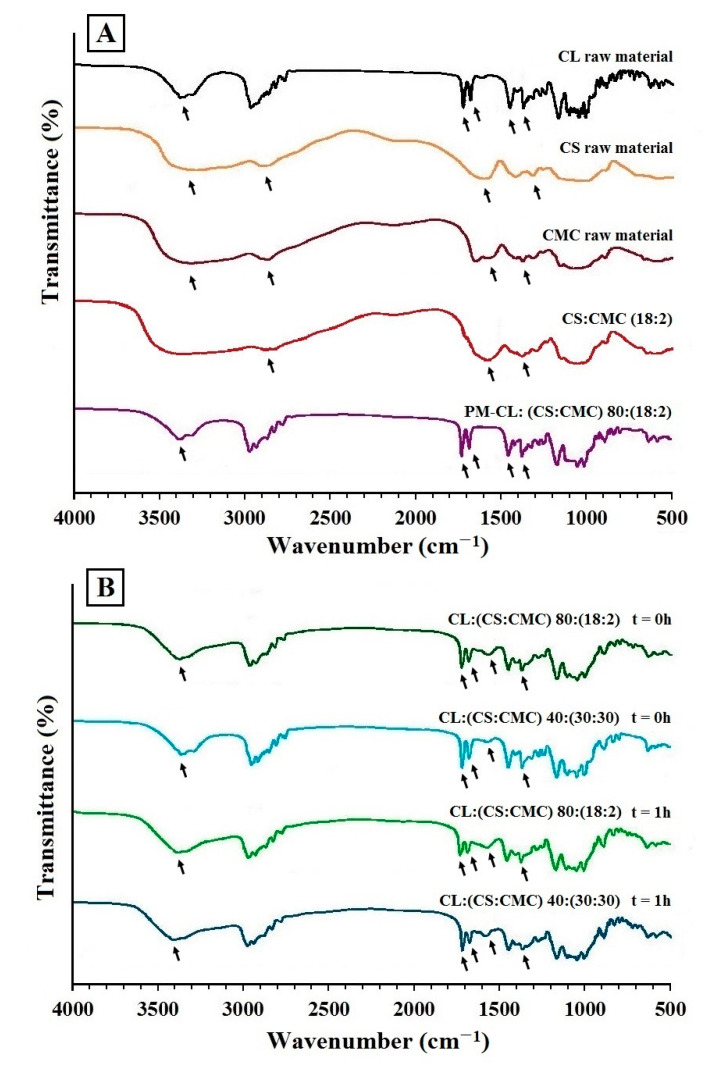
(**A**) TFIR spectrum of clarithromycin (CL), chitosan (CS), carboxymethyl cellulose (CMC), polyelectrolyte complex without clarithromycin CS:CMC (18:2), and physical mixture PM-CL:(CS:CMC) 80:(18:2), physical mixture PM-CL:(CS:CMC) 80:(18:2). (**B**) PEC tablets CL:(CS:CMC) 80:(18:2) and 40:(30:30) at 0 h and after 1 h in the dissolution medium (pH 4.2).

Figure 2 shows the FTIR of the raw materials (Figure 2A) clarithromycin (CL), chitosan (CS) carboxymethyl cellulose (CMC), physical mixture PM-CL:(CS:CMC) 80:(18:2) and PEC tablets CL:(CS:CMC) 80:(18:2) and 40:(30:30); these PEC tablets were analyzed at 0 h and after 1 h (Figure 2B) in the dissolution medium (acetate buffer, pH 4.2).

CL shows a broad band characteristic of O–H at 3394 cm^−1^ and the broad band between 2950 and 2800 cm^−1^ corresponding to alkyl-CH_3_ substitution bands (Figure 2A). Furthermore, the bands around 1733 and 1620 cm^−1^ are common for C=O stretching vibrations in the lactone ring, and the stretching vibrations at 1458 and 1372 cm^−1^ are related to C–CH_3_ and C–O bands [23,24]. The CS spectrum exhibits a broad band at 3394 cm^−1^ due to O–H stretching vibrations (Figure 2A), and characteristic bands around 1630 and 1365 cm^−1^ are observed for C=O stretching vibrations of amide I and amide III, respectively [2,5,25]. The CMC spectrum also presents a band at 3315 cm^−1^ corresponding to O–H stretching vibrations (Figure 2A), while the band at 2918 cm^−1^ is due to C–H stretching vibrations of the CH_2_ groups. The bands at 1620 and 1372 cm^−1^ correspond to the stretching vibrations of carboxylic and C–O groups [9,25]. The physical mixture CL:(CS:CMC) 80:(18:2) showed a broad band characteristic of O–H at 3394 cm^−1^, and the broad band between 2950 and 2800 cm^−1^ is due to alkyl-CH_3_ substitution bands (Figure 2A). The bands at 1733 and 1620 cm^−1^ correspond to C=O stretching vibrations in the lactone ring of CL, and the bands at 1457 and 1376 cm^−1^ correspond to the stretching vibrations of the C–CH_3_ and C–O bands.

The PEC CS:CMC (18:2) without CL, shown in Figure 2A, does not present bands corresponding to the stretching vibration in the lactone ring of CL and only presents bands at 1620 and 1372 cm^−1^, which correspond to the stretching vibrations of carboxylic and C–O groups [25].

In the PEC tablets of CL:(CS:CMC) 80:(18:2) and 40:(30:30) at the initial time (*t* = 0), the CL spectra, shown in Figure 2B, showed no changes in vibrations bands, indicating that there are no interactions between the CL and the polymeric complexes during the compression process. Similar results have been observed between PEC tablets and other active ingredients with carboxylic groups, such as azithromycin [24]. 

After 1 h in the dissolution medium (acetate buffer, pH 4.2), the PEC tablets CL:(CS:CMC) 80:(18:2) and 40:(30:30) showed a minor shifting of absorption bands at 1733 and 1620 cm^−1^, which correspond to O–C=O stretching vibrations in the lactone ring of CL. The characteristic bands around 1620 and 1372 cm^−1^ are due to the stretching vibrations of carboxylic and C–O groups corresponding to the CMC structure (Figure 2B) [5,25]. The absence of new bands in mixtures, in contrast to the individual polymers, confirm that no chemical reactions occurred between both polymers. The intensity of peaks could be correlated to the intensity of ionic interactions. In the dissolution medium, an interaction between the CS/CMC chains was produced, determining the formation of a stable ionic complex [26]. The polymer interactions indicated the formation of PEC between CS/CMC [2,8].

### 3.3. DSC Studies

**Figure 3:**
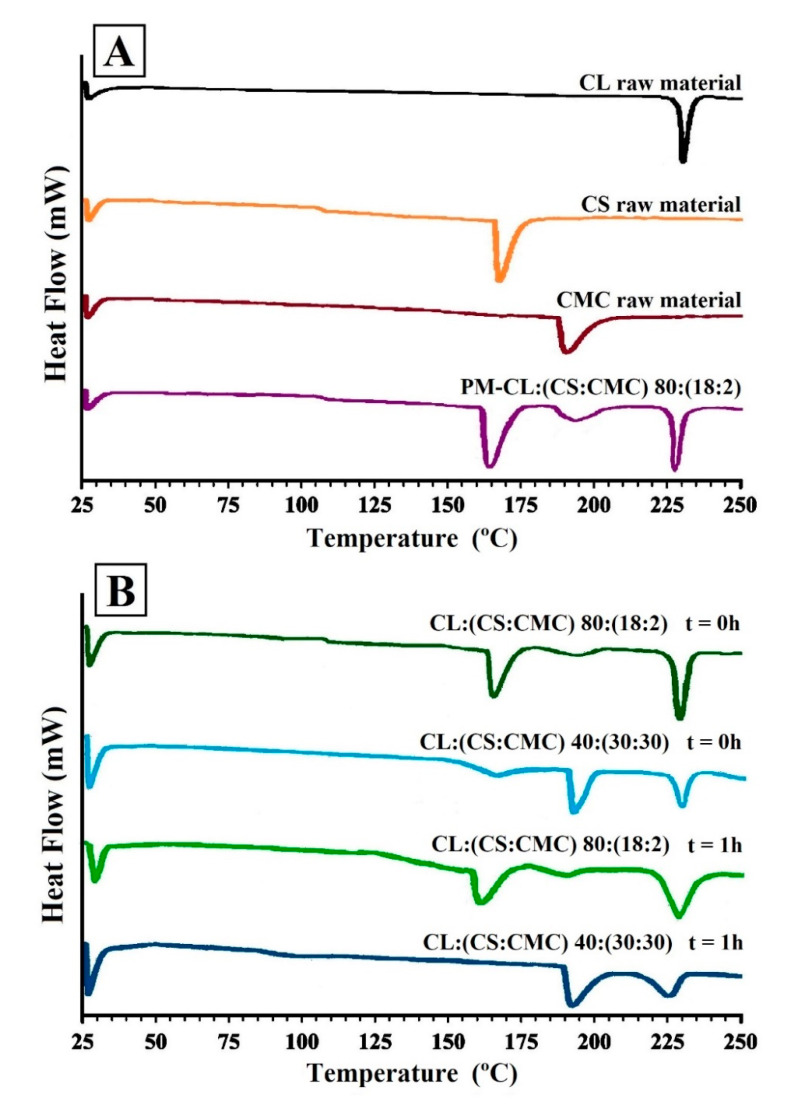
(**A**) DSC thermograms of clarithromycin (CL), chitosan (CS), carboxymethyl cellulose (CMC), and physical mixture CL:(CS:CMC) 80:(18:2). (**B**) PEC tablets CL:(CS:CMC) 80:(18:2) and CL:(CS:CMC) 40:(30:30) at 0 h and after 1 h in dissolution medium (pH 4.2).

Figure 3A shows the DSC scans of CL, CS, and CMC raw materials and the physical mixture CL:(CS:CMC) 80:(18:2). DSC scans of PEC tablets of CL:(CS:CMC) 80:(18:2) and 40:(30:30) at 0 h and these PEC tablets after 1 h in the dissolution medium are shown in Figure 3B.

The DSC of the CL raw material corresponds to the CL polymorph II (Figure 3A), characterized by an endothermic peak at 227.47 °C [27,28]. The CS exhibited (Figure 3A) an endothermic peak at 162.27 °C [8], and the CMC polymer at 191.46 °C [29]. The glass transition temperature (*T*_g_) for CS was observed between 100 and 110 °C [8,27]. *T*_g_ of CMC was not observed in the DSC curve due to the presence of intermolecular bonds in the cellulose chains that hindered its determination [29]. The physical mixture PM-CL:(CS:CMC) 80:(18:2) showed two endothermic peaks at 164.62 and 191.69 °C corresponding to CS and CMC, respectively (Figure 3A), and the endothermic peak at 228.13 °C corresponds to CL raw material. This physical mixture showed a *T*_g_ value for CS between 100 and 110 °C.

The thermograms of PEC tablets CL:(CS:CMC) 80:(18:2) and 40:(30:30) showed no significant differences in the melting temperatures of both polymers and CL, indicating that there are no drug/polymer interactions during the preparation process of PEC tablets [22]. The slight decrease in the CL endothermic peak and a small endothermic peak of CMC in CL:(CS:CMC) 80:(18:2) tablets compared to the physical mixture PM-CL:(CS:CMC) 80:(18:2) have been attributed to the formation of a PEC during tablet compression, indicating an entrapment of CL in the PEC [30]. The CL:(CS:CMC) 40:(30:30) showed a significant decrease corresponding to the CS melting peak due to the reduction of the polymer crystallinity after tablet compression with higher proportions of CS and CMC. These findings are consistent with the results observed in the FTIR studies.

After 1 h in the dissolution medium (acetate buffer, pH 4.2), the PEC tablets with low CS:CMC proportions, CL:(CS:CMC) 80:(18:2), showed a slight shift in the endothermic peaks of CS and CMC at 158.92 and 188.61 °C, respectively. The decrease in the melting peak of CS was due to CS/CMC interaction during the swelling process in the dissolution medium [31]. However, higher CS/CMC proportions in the complex CL:(CS:CMC) 40:(30:30) presented broad endothermic peaks of CMC and CL and a non-crystalline structure of CS owing to the formation of a polyelectrolyte complex in the dissolution medium [31]. Similar interpolymeric structures with CS have been previously observed [19].

### 3.4. Powder X-ray Diffraction (PXRD)

**Figure 4:**
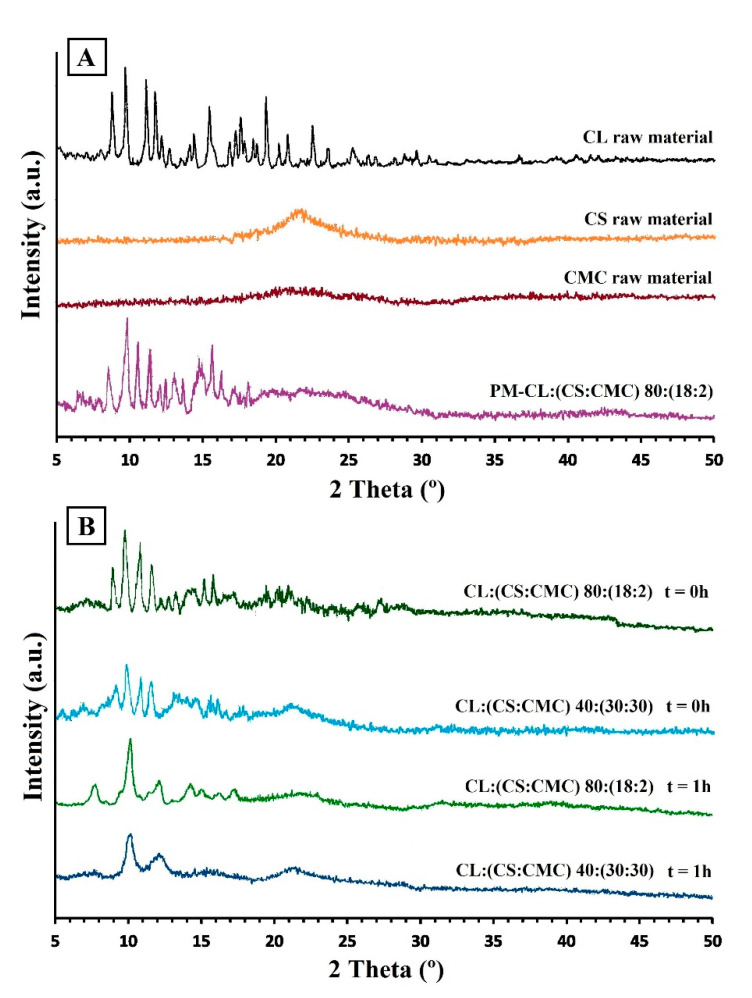
(**A**) PXRD of clarithromycin (CL), chitosan (CS), carboxymethyl cellulose (CMC), physical mixture of PM-CL:(CS:CMC) 80:(18:2). (**B**) PEC tablets CL:(CS:CMC) 80:(18:2) and CL:(CS:CMC) 40:(30:30) at 0 h and after 1 h in dissolution medium (pH 4.2).

The X-ray diffraction patterns in Figure 4A indicate a crystalline structure for CL raw material, with representative peaks at 8.54, 9.58, 10.83, and 11.56 °2θ. This crystalline structure corresponds to the CL form II [27]. The CS showed a broad semi-crystalline halo between 18 and 25 °2θ [32,33], while the CMC showed a small semi-crystalline halo between 18 and 26.5 °2θ, characteristic of cellulosic polymers [34]. The physical mixture PM-CL:(CS:CMC) 80:(18:2) shows peaks of CL raw material and a broad semi-crystalline halo between 18.5 and 26 °C corresponding to CS and CMC polymers (Figure 4A).

The X-ray spectra of the PEC tablets CL:(CS:CMC) 80:(18:2) and 40:(30:30) showed significant decreases in the intensity of the diffraction peaks of CL compared to the physical mixtures (Figure 4B). These results are due to entrapment of CL within the CS/CMC interpolymer complexes during the compression process [22]. The distribution of drugs within the PEC tablets induces a favorable energy effect compared to the physical mixture. In this approach, drug–chitosan interactions are able to control drug dissolution [32]. The lower intensity of the semi-crystalline halo of CS also indicates the formation of an ionic interaction between the cationic amino groups and the anionic carboxylic groups [5,33]. These results confirmed the formation of PEC observed in the DSC studies.

After 1 h in the dissolution medium (acetate buffer, pH 4.2), the PEC tablets CL:(CS:CMC) 80:(18:2) showed a diffraction peak at 10.06 °2θ due to the formation of a CL hydrate form within the PEC [27]. The CS/CMC semi-crystalline halo could not be observed in the X-ray spectra of the PEC tablets CL:(CS:CMC) 80:(18:2). However, the light semi-crystalline halo of CS between 18 and 26 °2θ observed in PEC tablets CL:(CS: CMC) 40:(30:30) indicates the possible formation of a hydrogel structure of CS around the interpolymer complexes during dissolution. Possibly, in these PEC tablets, the high proportion of CS used in the formation of the ionic double layer may mean that the CS chains are not available to form a hydrogel structure around the CS/CMC interpolymer complexes. Similar decreases in CS crystallinity have previously been observed due to the formation of PEC in the dissolution medium [32].

### 3.5. In Vitro Drug Release

The dissolution of CL from CS hydrogel tablets and different PEC CS/CMC complexes were studied in two different systems for representing gastric pH: 0.1 N HCl (simulated gastric fluid) and acetate buffer (pH 4.2).

Previous studies indicated that the highest solubility values of CL are obtained at a pH lower than 3.0 [15]. For this reason, we selected CS/CMC complexes with high CS ratios for dissolution studies in 0.1 N HCl, namely CL:(CS) 80:(20), CL:(CS:CMC) 40:(54:6), and 40:(30:30). CL:(CS) 80:(20) showed fast dissolution with a high percentage of CL at 30 min (90.64 ± 4.16%). The burst release for CL:(CS) 80:(20) has been attributed to the protonated amine groups of the CS chains in the dissolution medium [5,11]. The PEC tablets CL:(CS:CMC) 40:(54:6) and 40:(30:30) showed high dissolution percentages at 45 min (84.92 ± 3.68% and 82.28 ± 3.74%, respectively). High electrostatic repulsion between CS chains (pKa 6.5) and the significant relaxation of CMC polymer chains (pKa > 3.5) were observed in 0.1 N HCl simulated gastric fluid [9,13]. Moreover, a significant increase in the solubility of CL at low pH [11,15] could explain these fast dissolution profiles [4]. These immediate release systems in a gastric medium (0.1 N HCl) demonstrate rapid degradation of CL and are not suitable for gastric treatment of *Helicobacter pylori* [11].

The treatment of CL with omeprazole increases gastric pH to over 4.0 during 24 h [15]. The analysis of different CS/CMC complexes allows us to show different profiles of sustained CL release at pH 4.2. 

**Figure 5:**
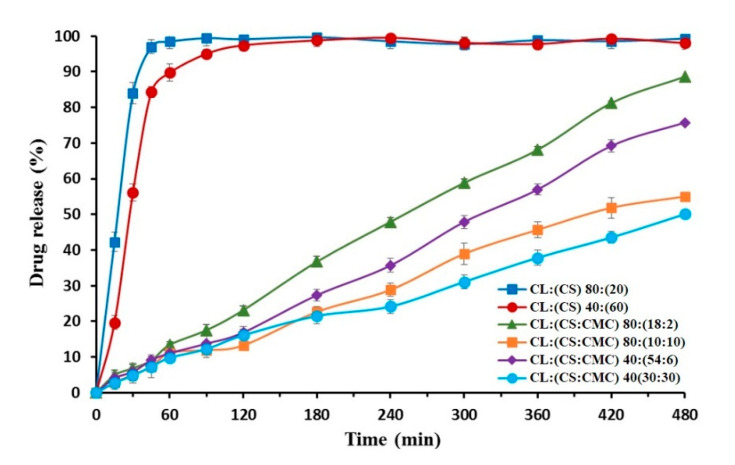
Release profiles of hydrogel tablets of CL:(CS) 80:(20) and 40:(60) and different PEC tablets of CL:(CS:CMC) 80:(18:2), 80:(10:10), 40:(54:6), and 40:(30:30) in acetate buffer (pH 4.2).

Figure 5 shows the CL release profiles obtained at pH 4.2 from hydrogel tablets of CL:(CS) 80:(20) and 40:(60) and different PEC tablets of CL:(CS:CMC) 80:(18:2), 80:(10:10), 40:(54:6), and 40:(30:30). The hydrogel tablets present a gel structure with a high dissolution percentage at 30 min (83.92 ± 4.62%) for CL:(CS) 80:(20), whereas the high CS proportion of CL:(CS) 40:(60) showed percentages of 56.12 ± 3.67% at 30 min. The burst release of the hydrogel structure CL:(CS) 80:(20) is attributed to the partially protonated amine groups of the CS chains in the dissolution medium (acetate buffer pH 4.2). The electrostatic repulsion of the cationic groups and high matrix swelling allow uptake in the dissolution medium [1]. This process results in fast-disintegrating hydrogel tablets that favor rapid drug release [4]. The kinetic parameters of these hydrogel tablets could not be evaluated due to their fast dissolution.

The PEC tablets with high proportions of chitosan, CL:(CS:CMC) 80:(18:2) and 40:(54:6), showed high dissolution percentages at 8 h (98.62 ± 4.06 and 75.76 ± 3.62%, respectively). The FTIR spectrum of these formulations at pH 4.2 revealed lower CS/CMC interaction and electrostatic repulsion between CS chains, which increases the water absorption and dissolution of CL. 

The PEC tablets with high proportions of carboxymethyl cellulose, CL:(CS:CMC) 80:(10:10) and 40:(30:30), showed a decrease in dissolution percentages at 8 h with values of 55.08 ± 1.46 and 50.07 ± 3.62%, respectively (Figure 5). The stronger interaction between CS/CMC resulted in low polymer swelling due to a greater attractive force between the polymer chains [5,33]. These sustained the release of CL for 6–8 h at pH 4.2 and are a suitable alternative for gastric treatment of *Helicobacter pylori* [11].

**Figure 6:**
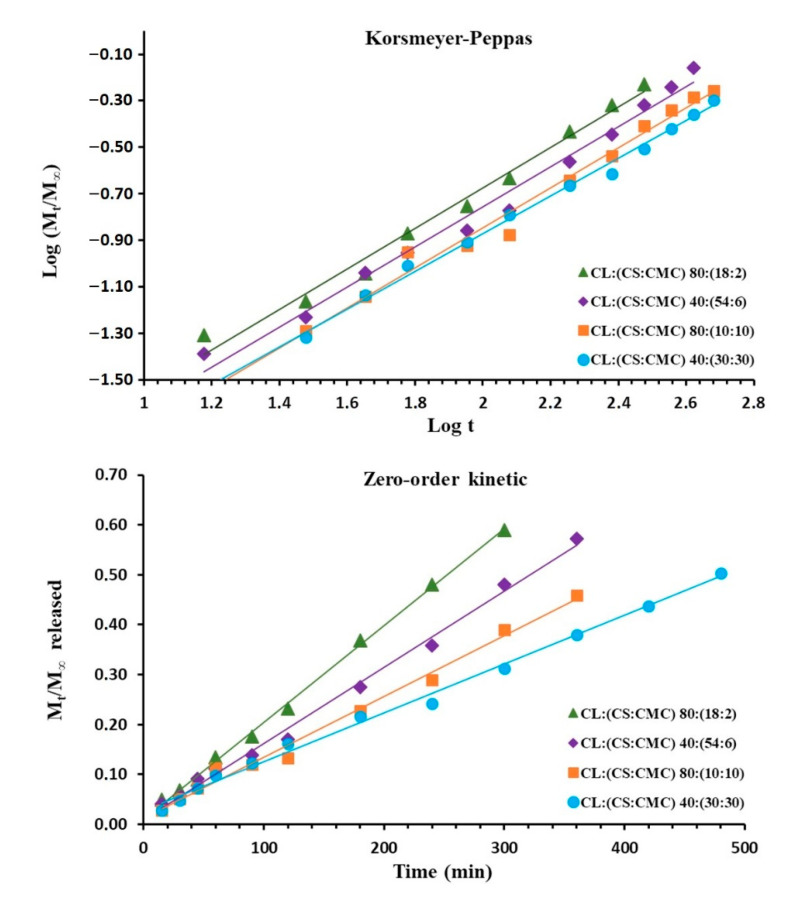
Korsmeyer-Peppas and zero-order kinetic models applied for clarithromycin release from PEC tablets CL:(CS:CMC) 80:(18:2), 40:(54:6), 80:(10:10), and 40:(30:30).

To evaluate the influence of CL within the PEC tablets, drug release kinetics were determined for the different PEC tablets. Figure 6 shows the Korsmeyer-Peppas and zero-order kinetic models of the PEC tablets CL:(CS:CMC) 80:(18:2), 40:(54:6), 80:(10:10), and 40:(30:30).

The Korsmeyer-Peppas kinetic model (Table 1) exhibits anomalous (non-Fickian) dissolution with *n* values close to 0.86, suggesting that the rate of water uptake into the matrix and CL release were controlled by diffusion through the hydrogel structure and swelling/erosion processes [16]. The absence of burst effects in the CS/CMC complexes justified the efficient interpenetration of CL within the network [35].

The zero-order kinetic model of PEC tablets with low CS/CMC interactions, CL:(CS:CMC) 80:(18:2) and 40:(54:6), showed the highest R^2^ values (0.9983 and 0.9944 respectively), followed by the Higuchi kinetic model (R^2^ values of 0.9629 and 0.9465), as presented in Table 2. The highest value of *K_0_* were found in CL:(CS:CMC) 80:(18:2) and 80:(54:6) with values of 0.0019 and 0.0015 min^−1^, respectively. These PEC tablets presented the formation of CS/CMC polyelectrolyte complexes that were surrounded by a large hydrogel layer formed by CS chains. These results indicate that CL release is controlled by rapid swelling and erosion processes from the CS/CMC hydrogel. These PECs showed kinetics of CL dissolution that fit Case II transport rather than non-Fickian transport (Table 1). The high *n* values in the Korsmeyer-Peppas equation indicate that in the dissolution medium, expansion of the CS chains is favored due to the repulsion between cationic charges, producing a hydrogel layer around the interpolymer complex [9]. Previous studies showed that the formation of a spherical hydrogel structure with *n* values > 0. 85 was also possible for Case II transport using the Korsmeyer-Peppas Equation [6,7].

The PEC tablets with high CS/CMC interactions, CL:(CS:CMC) 80:(10:10) and 40:(30:30), presented lower R^2^ values in the zero-order and Higuchi kinetic models (Figure 6 and Table 2). Previous studies showed that the optimal ratio of CS:CMC in the complex layer is 1:1 for sustained release [9]. Lower *K*_0_ values of CL:(CS:CMC) 80:(10:10) and 40:(30:30) were due to the higher CS/CMC interactions. The CL:(CS:CMC) 40:(30:30) showed the lowest values of *K*_0_ (0.0010 min^−1^) and *K_H_* (0.0254 min^−1^), which is due to the dense layer of polyelectrolyte between the cationic and anionic charged groups. These results correlate with the lowest *n* values (from 0.8611 and 0.8108) and fit for non-Fickian transport according to the Korsmeyer–Peppas equation (Table 1).

In CL:(CS:CMC) 40:(30:30), it is possible that the CS cationic groups are employed in the layer polyelectrolyte and that no free CS cationic groups are available to produce a hydrogel layer with cationic charges around the interpolymer complex [16]. Under these conditions, the entry of water will be delayed and the CL kinetics diminished. A similar delay in dissolution profiles was observed in PEC with CS as cationic polymer, with different proportions of xanthan gum or carboxymethyl starch as anionic polymers [8,33,35].

## 4. Conclusions

CS/CMC interpolymer complexes are good drug carriers for developing systems whereby drug release can be sustained for 8 h. During the compression process, intermolecular CS/CMC ionic interactions were observed via SEM, FTIR, DSC, and PXRD. According to SEM morphological studies, the different PEC tablets with CS/CMC exhibited changes in the density of their porous structures, which are related to the gel layer formed in the dissolution medium. The decrease in the crystallinity of CL was indicative of efficient interpenetration of CL within the interpolymer network. The FTIR studies of the PEC tablets in the dissolution medium (acetate buffer pH 4.2) showed a decrease in the amide groups of CS, which is related to the formation of the PEC.

Drug dissolution studies in acetate buffer (acetate buffer pH 4.2) indicate that the intermolecular interactions between CS/CMC play an important role in clarithromycin release kinetics. Specifically, the low CS/CMC interaction in CL:(CS:CMC) 80:(18:2) produced a significant expansion of the interpolymer chains with a kinetic model, indicating fast release, whereas high CS:CMC ratios in CL:(CS:CMC) 40:(30:30) resulted in a dense structure of PEC with a kinetic model that could be attributed to more sustained release and non-Fickian transport. These PEC tablets have great potential for use as a carrier in drug delivery systems.

## Figures and Tables

**Table 1:** Korsmeyer-Peppas kinetic model applied for clarithromycin release from PEC tablets CL:(CS:CMC) 80:(18:2), 40:(54:6), 80:(10:10), and 40:(30:30).

Formulations	*n*	R^2^
CL:(CS:CMC) 80:(18:2)	0.8696	0.9853
CL:(CS:CMC) 40:(54:6)	0.8613	0.9855
CL:(CS:CMC) 80:(10:10)	0.8611	0.9891
CL:(CS:CMC) 40:(30:30)	0.8108	0.9961

**Table 2:** Zero-order, Higuchi, and first-order kinetic models applied for clarithromycin release from PEC tablets CL:(CS:CMC) 80:(18:2), 40:(54:6), 80:(10:10), and 40:(30:30).

Formulations	Kinetic Models	*K*	R^2^
CL:(CS:CMC) 80:(18:2)	Zero-order	0.0019	0.9983
	Higuchi	0.0412	0.9629
	First-order	0.0084	0.9134
CL:(CS:CMC) 40:(54:6)	Zero-order	0.0015	0.9944
	Higuchi	0.0348	0.9465
	First-order	0.0070	0.9193
CL:(CS:CMC) 80:(10:10)	Zero-order	0.0012	0.9846
	Higuchi	0.0410	0.8634
	First-order	0.0056	0.8672
CL:(CS:CMC) 40:(30:30)	Zero-order	0.0010	0.9949
	Higuchi	0.0254	0.9717
	First-order	0.0052	0.8477

## Data Availability

Not applicable.
